# Might axial myofascial properties and biomechanical mechanisms be relevant to ankylosing spondylitis and axial spondyloarthritis?

**DOI:** 10.1186/ar4532

**Published:** 2014-04-08

**Authors:** Alfonse T Masi

**Affiliations:** 1 Department of Medicine, University of Illinois College of Medicine at Peoria, One Illini Drive, Peoria, IL 61656, USA

## Abstract

Ankylosing spondylitis and axial spondyloarthropathy have characteristic age- and sex-specific onset patterns, typical entheseal lesions, and marked heritability, but the integrative mechanisms causing the pathophysiological and structural alterations remain largely undefined. Myofascial tissues are integrated in the body into webs and networks which permit transmission of passive and active tensional forces that provide stabilizing support and help to control movements. Axial myofascial hypertonicity was hypothesized as a potential excessive polymorphic trait which could contribute to chronic biomechanical overloading and exaggerated stresses at entheseal sites. Such a mechanism may help to integrate many of the characteristic host, pathological, and structural features of ankylosing spondylitis and axial spondyloarthritis. Biomechanical stress and strain were recently documented to correlate with peripheral entheseal inflammation and new bone formation in a murine model of spondyloarthritis. Ankylosing spondylitis has traditionally been classified by the modified New York criteria, which require the presence of definite radiographic sacroiliac joint lesions. New classification criteria for axial spondyloarthritis now include patients who do not fulfill the modified New York criteria. The male-to-female sex ratios clearly differed between the two patient categories - 2:1 or 3:1 in ankylosing spondylitis and 1:1 in non-radiographic axial spondyloarthritis - and this suggests a spectral concept of disease and, among females, milder structural alterations. Magnetic resonance imaging of active and chronic lesions in ankylosing spondylitis and axial spondyloarthritis reveals complex patterns, usually interpreted as inflammatory reactions, but shows similarities to acute degenerative disc disease, which attributed to edema formation following mechanical stresses and micro-damage. A basic question is whether mechanically induced microinjury and immunologically mediated inflammatory mechanisms operate in both ankylosing spondylitis and degenerative disc disease but differ in relative degrees. The hypothesized biomechanical properties raised in this commentary require documentation of their association with the onset risk and course of ankylosing spondylitis and axial spondyloarthritis. If particular subsets of ankylosing spondylitis and axial spondyloarthritis patients are confirmed to have altered axial myofascial properties, their biological basis and underlying biomechanical mechanisms promise to become clarified. Understanding how biomechanical and physical properties can affect symptomatic and structural manifestations of these disorders could also improve their management.

## Background

This commentary updates our recent perspectives on integrated structural aspects of ankylosing spondylitis (AS) [1,2]. Axial myofascial hypertonicity is hypothesized to be a potential contributor to entheseal lesions via exaggerated attachment stresses [1,2]. Enthesopathy (Greek, *en* ‘in’ + *thesis* ‘a placing’ + *pathos* ‘suffering’) may be defined as an abnormal process occurring at insertion sites of muscle, tendons, and ligaments into bones or joint capsules [3]. These abnormalities often result from chronic mechanical overloading, with or without evidence of inflammation [3]. The novel hypothesis of a potential intrinsic myofascial mechanism is consistent with current enthesopathy concepts [1-3]. Recently, a murine model of spondyloarthritis (SpA) demonstrated that biomechanical stress and strain correlated with entheseal inflammation and new bone formation [4].

Myofascial tissues are integrated in the body into webs and networks, which permit transmission of passive and active tensional forces that provide stabilizing support and help to control movements. Passive or resting myofascial tension is the innate property, independent of central nervous system activation, which provides low-level stabilization to help maintain balanced postures [2]. Passive axial myofascial tension or tone is proposed to differ as a polymorphic trait among individuals in the population of varying age, sex, and possibly heredity, with minority subsets having counter-opposing outlier degrees of hypotonicity versus hypertonicity. Accordingly, axial myofascial hypertonicity was hypothesized to occur in a minority of individuals, which may predispose them to risk of AS [2].

## Pathophysiology of ankylosing spondylitis

Heritability of AS is greater than 90%, and numerous genetic markers have been identified in genome-wide association studies, but the pathophysiology remains poorly understood [5]. Chronic inflammation is present in the spinal fibrocartilaginous entheseal lesions of AS [3]. Synovitis and erosions also occur in the earlier stages of sacroiliac joint (SIJ) involvement and later evolve into the characteristic chondral fusion and bony ankylosis lesions [2,6]. Although mechanical stresses have been implicated in inflammation at entheseal sites [3,4] and in initiation of new bone formation or remodeling [4,7], the underlying basis for increased stresses and structural alterations in AS has not been defined.

A hypothesis of innate axial myofascial hypertonicity was derived as a possible contributor to increased biomechanical predisposition in AS [1,2,8]. The concept is consistent with the characteristic age- and sex-specific onset risk patterns of AS. The increased AS onsets during adolescent maturation of juveniles and in younger adulthood as well as in males versus females may reflect greater axial myofascial strengthening and stiffening in those respective subgroups [2]. The myofascial hypertonicity hypothesis also integrates form and force anatomical mechanisms, which can biomechanically link the characteristic spinal and SIJ lesions observed in AS [2,8,9]. For example, a stiffer axial myofascial system could chronically exaggerate tensional stresses and forces transmitted along the spine and might predispose patients to entheseal micro-damage and syndesmophyte formation [2,3]. Chronic spinal overloading could also predispose patients to enhanced SIJ micro-damage and repair pathways, leading to synovitis, erosions, and the later stages of enchondral ankylosis [2,6,8,9].

## Anti-tumor necrosis factor inhibitor therapy and structural progression in ankylosing spondylitis

The role of anti-tumor necrosis factor-blocking (anti-TNF-blocking) agents in modifying the inflammatory manifestations and new syndesmophyte formation in AS is incompletely understood [10-12]. Radiographic progression, as indicated by the modified Stokes AS Spinal Score (mSASSS), was analyzed in 22 patients with AS treated with infliximab over an 8-year follow-up as compared with 34 historical control AS patients who had never been treated with TNF blockers [10]. Although the mean (standard error of the mean) number of syndesmophytes was similar at baseline in the anti-TNF (3.6 ± 5.8) and the historical control (3.7 ± 1.0) patients, the rate of new syndesmophyte formation per patient over the 8 years was less (*P* = 0.007) in the infliximab (1.0 ± 0.6) versus historical control (2.7 ± 0.8) patients [10].

A more recent and larger observational study compared AS patients who had TNF-α inhibitor treatment with contemporaneous AS patients who had not been exposed (biologics-naïve) [11]. A reduced rate of interval radiographic progression (delta mSASSS) was found in the TNF group, but only for the treatment duration of 4 years or longer (relative rate 0.42, 95% confidence interval 0.18 to 0.98, *P* = 0.04). A protective effect of anti-TNF inhibitor therapy on new bone formation in AS is difficult to interpret because of complexities of the observational study designs [10,11]. However, the data suggest that anti-TNF agents do not accelerate its structural progression [12]. Further assessment of inflammatory and biomechanical influences on the clinical and structural progression of AS and axial SpA is needed [12,13].

## Host relations to the onset and course of ankylosing spondylitis

Historically, AS has been classified by the modified New York criteria which require the presence of definite radiographic SIJ lesions [14]. More recently, the Assessment of SpondyloArthritis international Society had developed classification criteria for axial spondyloarthritis (axSpA) [15], which include patients with non-radiographic (undifferentiated) axSpA (nr-axSpA) who do not fulfill the modified New York criteria [14].

More accurate differentiation of patients satisfying the respective AS [14] and nr-axSpA [15] classification criteria promises to enhance earlier diagnosis, treatment trials, and improved prediction of outcomes [14-17]. For example, the male-to-female sex ratios clearly differed between the two patient categories: 2:1 or 3:1 in AS and 1:1 in nr-axSpA [16,17]. A milder form of AS severity, observed predominantly in females, had previously been proposed for HLA-B27-positive relatives of AS probands who had characteristic symptoms but not radiographic evidence of SIJ or spine abnormalities [18]. A spectral concept of AS [15-18] would also warrant further studies of biomechanical influences affecting host predisposition to AS vis-a-vis nr-axSpA.

## Magnetic resonance imaging of active and chronic lesions in ankylosing spondylitis and axial spondyloarthritis

Mechanisms causing signal changes in magnetic resonance imaging (MRI) images of the spine and SIJs are complex and interpretation has varied [10,17,19,20]. In degenerative disk disease (DDD), acute or Modic type 1 changes (increased T2-weighted and decreased T1-weighted images) of vertebral endplate bone and marrow are believed to represent edema from vascularized fibrous tissue resulting from mechanical stresses and micro-damage [19]. In AS and axSpA, similar acute MRI signal abnormalities localized characteristically at entheseal vertebral edges (VEs) are interpreted as inflammatory lesions [10,17,20]. Modic type 1 lesions in both DDD and AS are associated with pain as well as inflammatory mediators found localized to such acute MRI changes [17,19].

The later-stage Modic type 2 MRI changes (increased T1-weighted and iso- to hyper-intense T2-weighted images) reflect fatty degeneration of the bone marrow, as is observed in both DDD [19] and AS [20]. The latest-stage Modic type 3 change (decreased T1- and T2-weighted images) is also observed in both diseases and is attributed to radiographically demonstrated sclerosis and bone formation of osteophytes in DDD [19] and syndesmophytes in AS [20]. A basic question is whether mechanically induced microinjury and immunologically mediated inflammatory mechanisms operate in both AS and DDD but to different relative degrees.

The relation of sequential type 1 to type 3 changes to the frequency of new syndesmophyte formation at VEs was investigated in 73 patients with AS treated with TNF-blocking agents [20]. If the baseline inflammatory (type 1) changes resolved during therapy, no new syndesmophyte formation was observed in follow-up [20]. In contrast, if baseline fatty degeneration (type 2) lesions remained unchanged under anti-TNF therapy at 2 years, then the relative risk for new syndesmophyte formation from baseline to 2 years was 5.0 (*P* = 0.002) and to 5 years was 3.3 (*P* = 0.009) [20]. The underlying bases of early and sequential MRI changes in AS and axSpA deserve further definition and differentiation.

If the novel biomechanical properties raised in this commentary are documented to associate with predisposition or course of AS and axSpA, then their underlying mechanisms could help to clarify the biological basis, classification, and management of these disorders and to improve understanding of how physical modalities can affect symptomatic and functional measures.

## Abbreviations

AS: Ankylosing spondylitis; axSpA: Axial spondyloarthritis; DDD: Degenerative disk disease; MRI: Magnetic resonance imaging; mSASSS: Modified Stokes Ankylosing Spondylitis Spinal Score; nr-axSpA: Non-radiographic (undifferentiated) axial spondyloarthritis; SIJ: Sacroiliac joint; SpA: Spondyloarthritis; TNF: Tumor necrosis factor; VE: Vertebral edge.

## Competing interests

The author declares that he has no competing interests.
